# Supplementary material for: The interactome of intact mitochondria by cross-linking mass spectrometry provides evidence for coexisting respiratory supercomplexes
Source: Mol Cell Proteomics. 2017 Dec 8;17(2):216–32. doi: 10.1074/mcp.RA117.000470 (PMC5795388; doi:10.1074/mcp.RA117.000470)
Supplement: Supplemental Data [file supp_RA117.000470_133922_0_supp_23978_fzffwf.zip › spectra_annotation/mito_spectra_annotation/269-1-6-1-7-1.pdf]

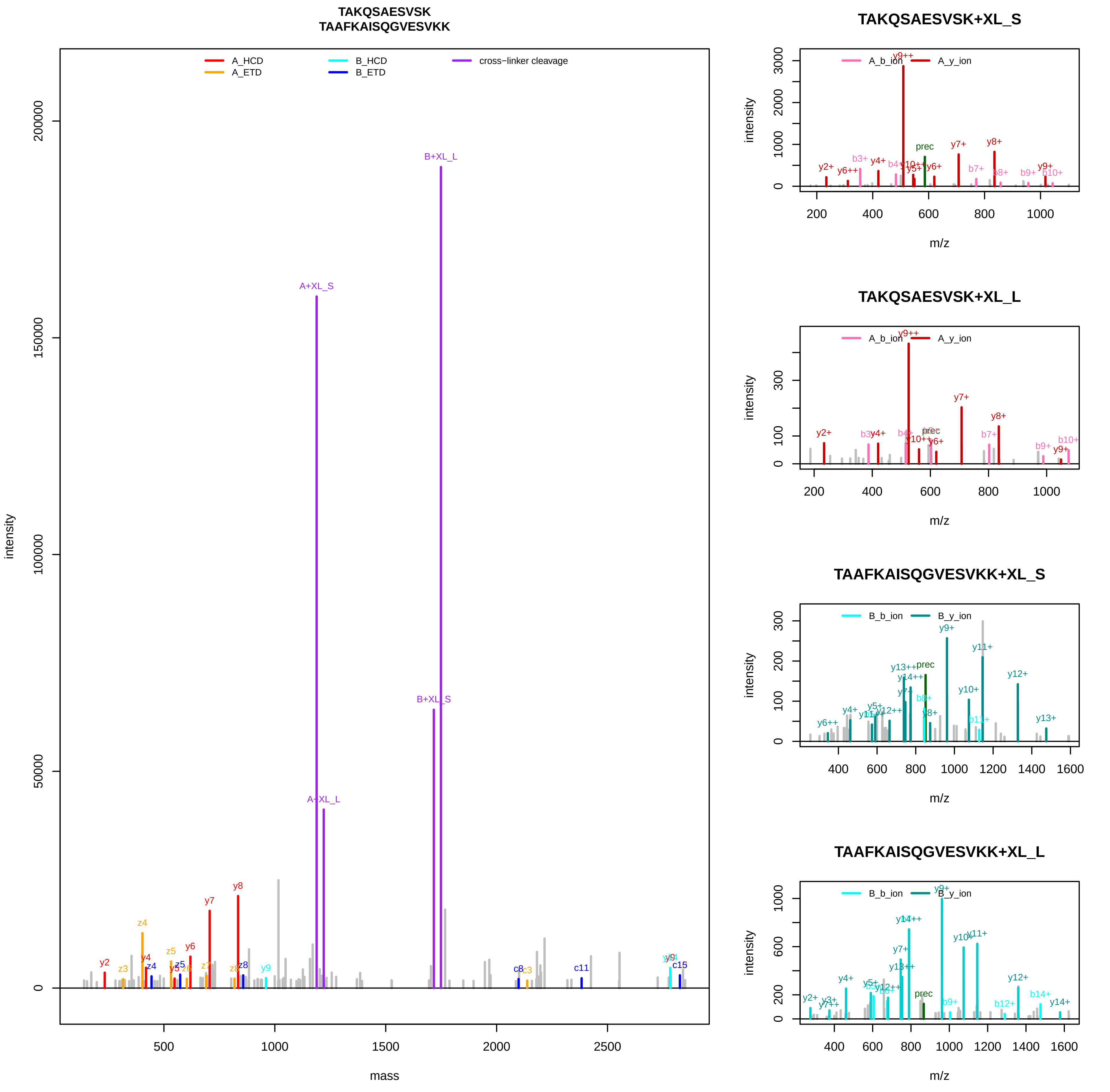
TAKQSAESVSK
TAAFKAISQGVESVKK
A_HCD
A_ETD
B_HCD
B_ETD
cross−linker cleavage
B+XL_L
A+XL_S
B+XL_S
A+XL_L
y2
z3
z4
y4
z4
z5
y5
z5
z6
y6
z7
y7
z8
z8
y8
y9
c8
c3
c11
y9
c15
intensity
mass
0
50000
100000
150000
200000
500
1000
1500
2000
2500
TAKQSAESVSK+XL_S
A_b_ion
A_y_ion
y9++
prec
y7+
y8+
y2+
y6++
b3+
y4+
b4+
y10++
y5+
y6+
b7+
b8+
b9+
y9+
b10+
intensity
m/z
0
1000
2000
3000
200
400
600
800
1000
TAKQSAESVSK+XL_L
A_b_ion
A_y_ion
y9++
y7+
y8+
y2+
b3+
y4+
b4+
prec
y10++
y6+
b7+
b9+
y9+
b10+
intensity
m/z
0
100
300
200
400
600
800
1000
TAAFKAISQGVESVKK+XL_S
B_b_ion
B_y_ion
y9+
y11+
y13++
prec
y14++
y12+
y10+
y7+
b8+
y5+
y12++
y4+
y6++
y8+
b11+
y13+
intensity
m/z
0
100
200
300
400
600
800
1000
1200
1400
1600
TAAFKAISQGVESVKK+XL_L
B_b_ion
B_y_ion
y9+
y14++
y10+
y11+
y7+
y13++
y4+
y5+
y12++
y2+
y3+
y7++
prec
b9+
y12+
b12+
b14+
y14+
intensity
m/z
0
200
600
1000
400
600
800
1000
1200
1400
1600
